# Single-cell RNA sequencing of adenoid cystic carcinoma of the breast reveals cellular heterogeneity and tumor microenvironment features

**DOI:** 10.1186/s12920-026-02329-2

**Published:** 2026-02-27

**Authors:** Zhenning Tang, Jufen Zhao, Xiaoying Huang, Qingyuan Liu, Yuchen Wang, Chaolin Zhang, Ling Li

**Affiliations:** 1 Department of Surgical Oncology, General Hospital of Ningxia Medical University, Yinchuan, 750004 China; 2 Clinical Laboratory, The First People’s Hospital of Yinchuan, Yinchuan, 750010 China; 3 Department of Neurology, The First People’s Hospital of Yinchuan, Yinchuan, 750001 China

**Keywords:** ScRNAseq, Adenoid cystic carcinoma of the breast, Tumor microenvironment, H19+ Myoepithelial Cells

## Abstract

**Background:**

Adenoid cystic carcinoma of the breast (ACCB) is a rare malignant tumor with distinct pathological features and a relatively favorable prognosis compared to other breast cancer subtypes, yet its molecular mechanisms remain poorly understood due to challenges in sample acquisition and targeted research.

**Methods:**

In this study, we employed single-cell RNA sequencing (scRNA-seq) to systematically characterize the cellular heterogeneity and tumor microenvironment in ACCB. We obtained scRNAseq data from a single patient with ACCB and integrated it with publicly available datasets from head and neck adenoid cystic carcinoma (HNACC) and triple-negative breast cancer (TNBC). Through high-resolution gene expression profiling, cell clustering, differential expression analysis, and cell-cell communication analysis, we aimed to identify key malignant subpopulations and regulatory networks in ACCB.

**Results:**

Unsupervised clustering revealed seven major cell types in the analyzed ACCB sample, including epithelial cells, fibroblasts, and immune cells. In-depth analysis of epithelial cells identified H19 + myoepithelial cells (H19 + myoEpC) as the dominant malignant subpopulation, enriched in ACCB compared to other cancers and characterized by high expression of oncogenic pathways and ligands such as LAMB1 and WNT6. Tumor-associated fibroblasts exhibited significant heterogeneity, with cancer-associated fibroblasts (CAFs) expressing EMT-related genes and interacting closely with H19 + myoEpC. The tumor microenvironment was immunosuppressive, with reduced infiltration of CD4 + and CD8 + T cells and B cells, and lacked classical Treg/Tex populations. Comprehensive cell-cell communication analysis showed that H19 + myoEpC orchestrated a signaling network with CAFs, immune cells, and endothelial cells, driving tumor progression and immune evasion.

**Conclusions:**

Our study provides the single-cell resolution map of ACCB from a representative case, highlighting the unique role of H19 + myoEpC in tumor progression and immune suppression. These findings offer new insights into the molecular classification of ACCB and potential therapeutic targets.

**Supplementary Information:**

The online version contains supplementary material available at 10.1186/s12920-026-02329-2.

## Introduction

ACCB is a rare malignant breast tumor with distinct pathological characteristics. In recent years, with the continuous advancement of diagnostic techniques for breast diseases, the detection rate of ACCB has increased, though its incidence remains relatively low, accounting for approximately 0.06 to 0.1% of all breast cancer [1, 2]. Histologically, ACCB resembles salivary gland adenoid cystic carcinoma and is characterized by pseudoglandular or cribriform structures formed by basaloid and luminal cells [3]. The molecular subtype of ACCB predominantly corresponds to TNBC. However, its clinical characteristics and prognosis markedly differ from those associated with other pathological variants of TNBC [4]. TNBC typically has a poorer prognosis, but the ten-year survival rate for patients with ACCB surpasses 90% [5]. ACCB has a distant metastasis rate of less than 20%, typically involving the lungs and bones [6]. Compared to other breast cancer subtypes, research on the molecular mechanisms of ACCB has long been delayed, primarily due to challenges in sample acquisition caused by its rarity, insufficient clinical awareness, and the lack of a targeted molecular classification system [7]. This knowledge gap has significantly hindered the development of precision diagnostic and therapeutic strategies. However, the unique pathological features and clinical manifestations of ACCB suggest that it may exhibit distinct biological behaviors and treatment responses compared to other breast cancer types. Therefore, in-depth research into the pathogenesis, diagnostic approaches, and treatment strategies for ACCB is of critical clinical importance for improving patient prognosis and survival rates.

Previous studies utilizing whole-exome sequencing and transcriptome analyses have identified key molecular features of ACC, including the high-frequency MYB-NFIB gene fusion event and aberrant activation of Notch and PI3K/AKT/mTOR signaling pathways [8, 9]. However, these findings were predominantly derived from bulk sequencing techniques at the tissue level, which fail to resolve intratumoral cellular heterogeneity or dynamic microenvironmental interactions. Furthermore, current investigations into ACCB drug resistance mechanisms and immune microenvironment featuresremain substantially limited, resulting in a critical lack of predictive biomarkers and effective therapeutic targets in clinical practice. The groundbreaking development of scRNA-seq technology has provided unprecedented resolution for deciphering tumor heterogeneity, microenvironment remodeling, and mechanisms of therapy resistance [10]. In breast cancer research, scRNA-seq has been successfully applied to subtyping TNBC, dissecting mechanisms of immune checkpoint inhibitor resistance, and dynamically monitoring pre-metastatic niche formation [11, 12]. Recent studies have employed scRNA-seq to elucidate the tumor cell states and epigenetic features associated with endocrine therapy resistance in breast cancer [13]. Additionally, another study utilized single-cell RNA sequencing and multi-omics analysis to uncover a significant correlation between cholesterol biosynthesis and HER2 expression, proposing a therapeutic strategy targeting both pathways [14]. These investigations not only reveal the metabolic reprogramming mechanisms of breast cancer cells during treatment but also provide crucial mechanistic insights for the rational design of combination therapies. While single-cell technologies have demonstrated tremendous value in oncology research, their application to the ACCB remains notably limited.

In this study, we innovatively employ scRNA-seq to systematically characterize cellular heterogeneity in breast adenoid cystic carcinoma. Through high-resolution gene expression profiling, we aim to unravel its complex pathogenesis and identify potential therapeutic targets, thereby establishing both theoretical foundations and practical directions for precision medicine in this rare malignancy.

## Materials and methods

### Data collection

The single-cell transcriptomic data of HNACC (GSE210171) and TNBC (GSE176078) used in this study were obtained from the GEO database (https://www.ncbi.nlm.nih.gov/geo/). The tumor tissues and adjacent normal tissues from one patient, who was a 46-year-old woman diagnosed as an ACCB patient, were obtained from the General Hospital of Ningxia Medical University. The studies involving human participants were reviewed and approved by the ethics committee. The patients provided written informed consent to participate in this study.

### 10x Genomics Chromium library construction and sequencing

A 10x Genomics Chromium machine was used for > 10,000 single-cell capture and cDNA preparations according to the Single Cell 3’ Protocol recommended by the manufacturer. Chromium Single Cell 3’ Library and Gel Bead Kit V3.1 (10× Genomics, PN1000268) was used to generate single-cell gel beads in emulsion (GEM). The captured cells were lysed, and the released RNA was reverse-transcribed with primers containing poly-T, barcode, unique molecular identifiers (UMIs), and read 1 primer sequence in GEMs. Barcoded cDNA was purified and amplified by PCR. The adapter ligation reaction was performed to add the sample index and the read 2 primer sequence. After quality control, the libraries were sequenced on the Illumina Novaseq platform in 150 bp pairen-end manner.

### Quality control and doublets removal

The Cell Ranger toolkit (10x Genomics, v7.1.0) was utilized to align reads to the GRCh38-2020-A reference genome and generate the matrix of genes versus cells. To filter low quality cells and enrich for high quality cells in each dataset, Quality control and doublet removal were performed for each dataset individually. Further quality control of each cell was applied as follows: (1) The number of unique molecular identifier (UMI) counts > 1000; (2) The number of detected genes > 500; (3) The proportion of mitochondrial gene expression < 25%; (4) Exclusion of cells predicted as doublets by DoubletFinder. A total of 29,435 cells were included in further downstream analysis.

### Cell clustering and dimensionality reduction

The Seurat package (version 3.1.5) was used to perform downstream analysis and visualization [15]. Specifically, the NormalizeData function was used to normalize the raw counts with LogNormalize, and the scale factor set to 10,000. Find VariableFeatures was used to identify highly variable genes (HVGs) for each dataset. The top 2,000variable genes were summarized by principal component analysis(PCA) into 30 principal components (PCs), and the cells were visualized in a two-dimensional Uniform Manifold Approximation and Projection (UMAP). Cell clusters were identified using the FindClusters with a resolution of 1 from Seurat, based on a Shared Nearest Neighbor (SNN) graph.

### Unsupervised clustering and cell type annotation

Further analysis was performed with Seurat (v4.1.0) following the recommended pipeline (https://satijalab.org/seurat/). Cell cycle scores were assessed using the “Cell CycleScoring” function in Seurat [16]. The top 3000 highly variable genes were identified for PCA. To account for batch effects across different libraries, Harmony (v1.0) was used to remove them. The top 30 PCs were then selected for further dimension reduction. UMAP was performed. Cell clusters were identified using Seurat’s the FindClusters function with the default resolution parameter. Cluster-specific marker genes were detected using FindAllMarkers function with default settings. Cell identities were assigned by comparing cluster-enriched marker genes with canonical markers from the literature and the CellMarker database.

### Differential analysis for clusters

The FindAllMarkers function implemented in Seurat was used to identify DEGs between different clusters [17]. We set the parameter “only.pos” as “TRUE,” which returned only the genes expressed at a significantly higher level in a given cluster. The Wilcoxon test was performed on each gene (Only genes that are detected in a minimum fraction of 0.25 cells in either of two populations were included for the test), and the P value and adjusted P value, standing for statistical, significance were computed. Significant DEGs were selected from genes with P_val_adj ≤ 0.01 and avg_logFC ≥ 0.5.

### Identification of cancer cells

To identify malignant and non-malignant cells, we confidently distinguished between them in each sample using two complementary approaches. First, we identified malignant epithelial cells using the marker genes *EPCAM*, *KRT18*, *KRT14*, and *KRT19*. Somatic large-scale chromosomal CNV score of each ductal cell was calculated using the R package inferCNV (v1.10.1). A raw counts matrix, an annotation file, and a gene/chromosome position file were prepared according to the data requirements (https://github.com/broadinstitute/inferCNV). Epithelial cells were used as the observation cells, and B cells, T cells, myeloid cells, endothelial cells, fibroblasts, and pericytes were used as reference cells. All default parameters were used except cutoff = 0.1 and denoise = TRUE. Using hierarchical clustering of copy number variation (CNV) profiles, we identified a subset of cells with large-scale CNV on chromosome 17 as malignant.

### The regulon activity of TFs with SCENIC

To dissect transcriptional regulatory programs in epithelial subtypes, we performed single-cell regulatory network inference and clustering (SCENIC) (v1.3.1) analysis with default parameters. First, co-expression modules between transcription factors (TFs) and potential target genes were inferred using GENIE3 (v1.16.0). Subsequently, TF binding motif enrichment was evaluated using RcisTarget (v1.14.0) with the motif database (hg38 v10), where regulatory regions were defined as either ± 10 kb around the transcription start site (TSS) or 500 bp upstream of the TSS. Regulons—comprising a TF and its high-confidence target genes—were reconstructed by integrating co-expression and motif enrichment results. Regulon activity per cell was quantified using AUCell (v1.16.0), which calculates the area under the recovery curve (AUC) to assess the relative enrichment of regulon target genes in each cell’s expression ranking. The resulting AUCell score matrix was then used to compute regulon specificity scores (RSS) for each epithelial subtype using the calcRSS function. Finally, subtype-specific regulons were visualized using ggplot2 (v3.3.5).

### Non-negative matrix factorization (NMF)

To identify gene-expression programs characteristic of epithelial subtypes, we performed non-negative matrix factorization using the R package NMF (v0.23.0). First, we selected 6000 highly variable genes using the “vst” method and scaled the expression values. The scaled data matrix was then converted to a non-negative matrix by setting all negative values to zero, as required for NMF. Factorization was performed using the “snmf/r” algorithm with a rank of 10, yielding 10 metagenes (expression programs) and their corresponding coefficient matrices. For each metagene, the top 50 genes with the highest loadings were extracted. This process identified 10 gene-expression programs corresponding to distinct transcriptional patterns across epithelial subtypes.

### Gene enrichment analysis

The FindMarkers (Wilcoxon test) function in Seurat was used to identify differentially expressed genes across comparisons. GO and KEGG enrichment analysis were performed by the ClusterProfiler package (v4.2.2) for differentially expressed genes in each cluster [18]. An adjusted Padj <0.05 was used as the screening criterion for significant enrichment in all enrichment analyses. GSEA was performed to identify which gene sets were significantly enriched in each cell cluster. Only gene sets with false discovery rate (FDR) *p* values < 0.05 and nominal p values < 0.05 were considered significantly enriched.

### Cell–cell communication analysis

A cell communication analysis was performed using the R package CellChat (v1.4.0) [19]. The “CellchatDB.human” database was set as the ligand-receptor interaction database. Overexpressed signaling genes and ligand-receptor interactions (pairs) were identified using the “identifyOverExpressedGenes” and “identifyOverExpressedInteractions” functions. The communication probability between any interaction cell groups with a minimum cell count of 10 was calculated using the “computeCommunProb” function. By summarizing all related ligands and receptors, the communication probability at the signaling pathway level was calculated using the “computeCommunProbPathway” function. The ligand-receptor pairs with a *P* value < 0.05 were retained.

## Results

### Single-cell transcriptomic profiling identifies seven major cell types in ACCB

**Fig. 1:**
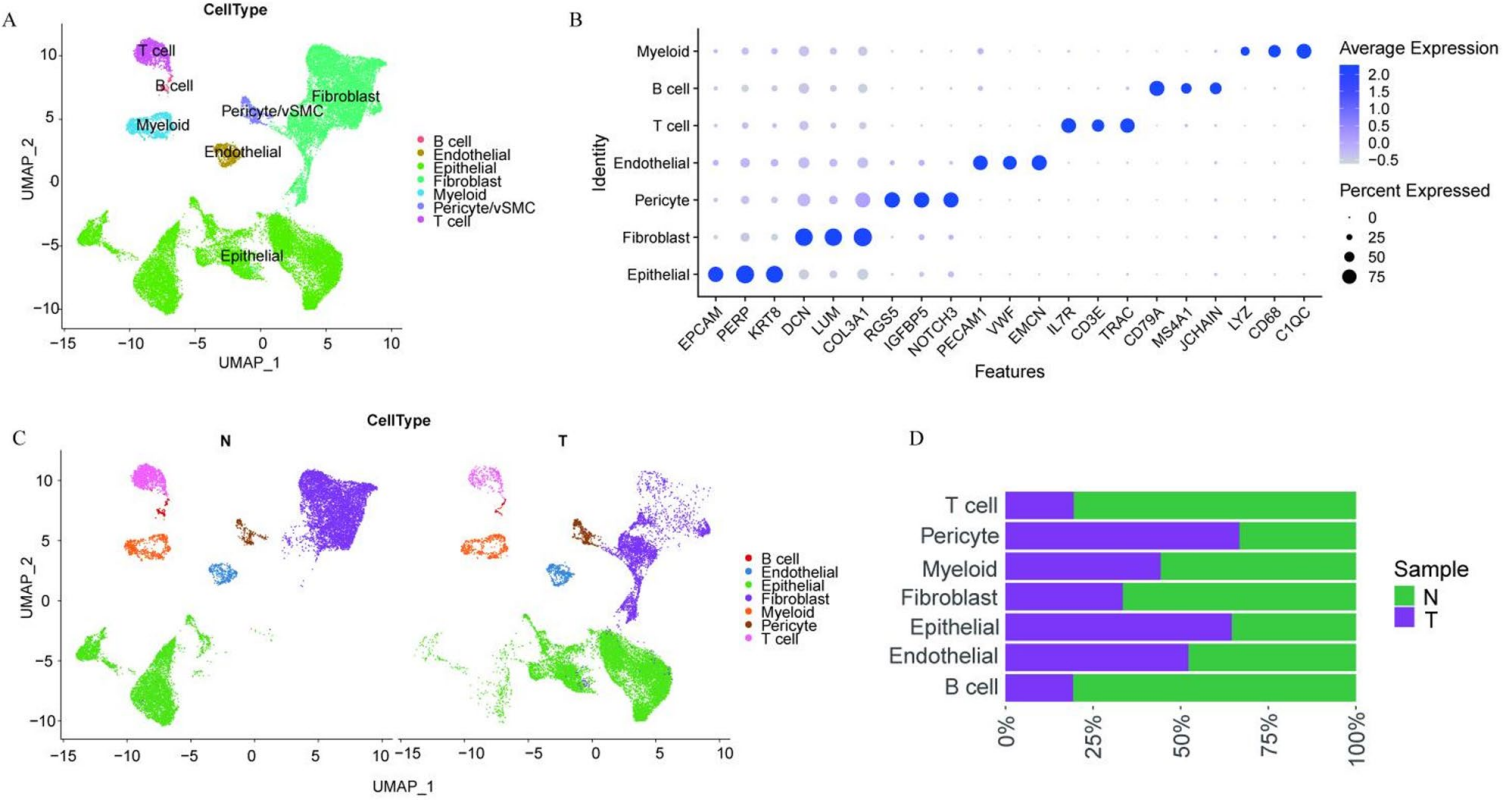
Landscape of single cells derived from ACCB and adjacent normal tissues. **A** UMAP plot showing seven major cell clusters identified from integrated scRNA-seq data of ACCB tumors and adjacent normal tissues. Each cluster is annotated based on canonical marker expression. **B** Dot plot visualizing the expression levels of key marker genes used for cell type annotation. Dot size indicates the percentage of cells expressing the gene within each cluster, and color intensity represents the average expression level. **C** UMAP plot showing the distribution of seven distinct cell subtypes in ACCB (T) and adjacent normal tissues (N). **D** Bar plot comparing the cellular composition between tumor (T) and adjacent normal tissues (N), illustrating the relative abundance of each cell type

We performed unsupervised clustering and dimensionality reduction on scRNA-seq data from tumor and adjacent normal tissues of a single ACCB patient using the Seurat package. Initial quality control filtered genes expressed in ≥ 3 cells and cells with ≥ 200 detected genes. Further refinement retained cells with 200–8,000 expressed genes and < 20% mitochondrial gene content. After normalization, 2,000 highly variable genes were selected for dimensionality reduction, revealing seven major cell clusters: B cells, endothelial cells, epithelial cells, fibroblasts, myeloid cells, pericytes/vSMCs, and T cells (Fig. 1A, B). Comparative analysis of tumor and adjacent normal tissues demonstrated distinct distribution patterns and phenotypic features across cell clusters (Fig. 1C, D). The results showed that T cells, B cells, and fibroblasts were more abundant in adjacent normal tissues, whereas epithelial cells, endothelial cells, and pericytes were more abundant in tumor tissues.

### Analysis of epithelial cell subtypes identifies H19 + MyoEPC as the dominant malignant subpopulation

**Fig. 2:**
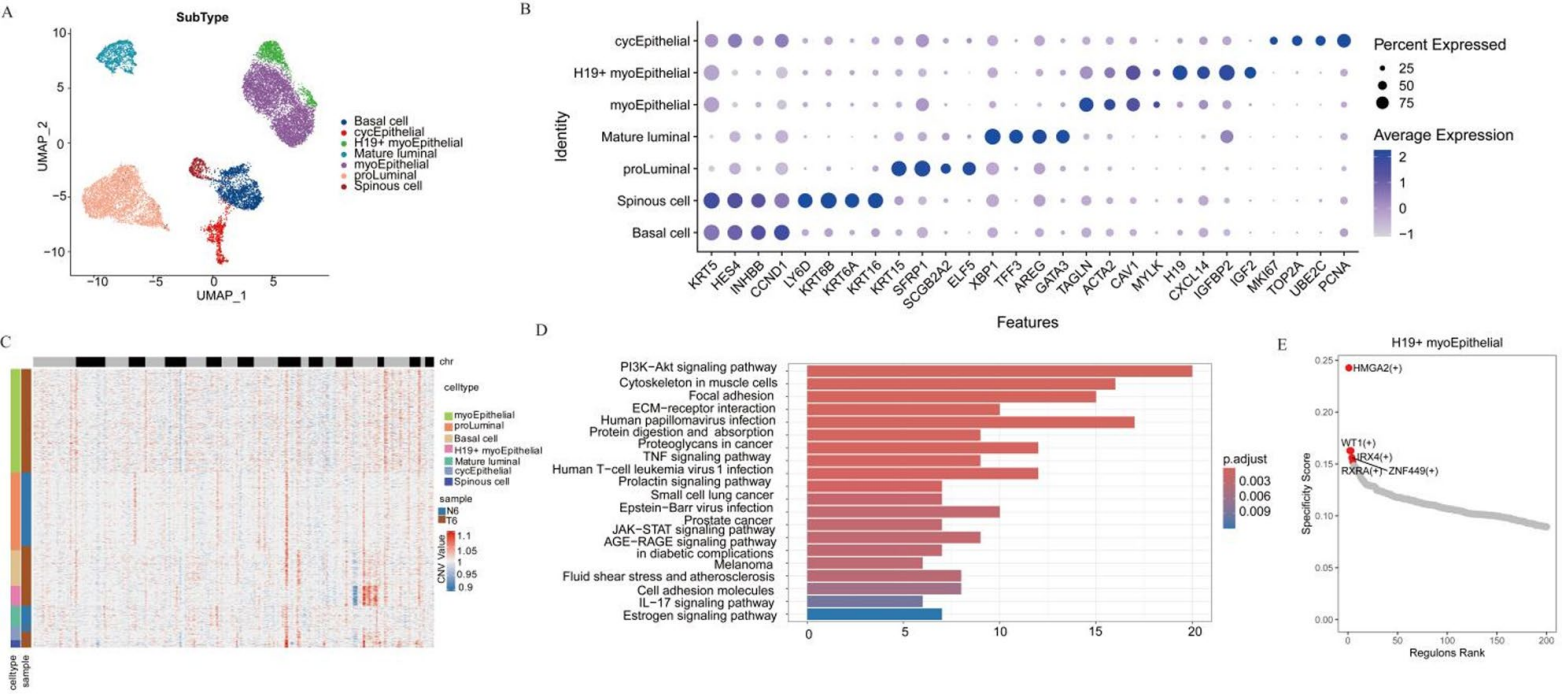
Analysis of the epithelial subtypes in the single-cell transcriptome. **A** UMAP plot showing seven transcriptionally distinct subclusters identified from epithelial cells. **B** Dot plot showing the expression of specific marker genes across different epithelial cell subtypes. **C** Heatmap illustrating the CNV in epithelial cells, with H19 + myoEPC showing the majority of CNVs, indicating a predominant malignant subpopulation. **D** KEGG analysis on the differential genes among subtypes. **E** Gene regulatory network analysis identifying four critical TFs (HMGA2, WT1, IRX4, and RXRA) associated with H19 + myoEPC

In-depth analysis of epithelial cells identified seven distinct subclusters (Fig. 2A). Based on the expression of relevant marker genes, these subclusters were defined as basal cells, cycling epithelial cells (cycEpithelial), H19 + myoEPC, mature luminal cells, myoepithelial cells, pro-luminal cells, and spinous cells (Fig. 2B). Using the “interCNV” package, large-scale CNVs were analyzed with myeloid cells as a control, enabling the classification of epithelial cells into malignant and non-malignant populations. The results revealed that the majority of CNVs occurred in H19 + myoEPC (Fig. 2C and Supplementary Fig. S1). Differential gene expression analysis of epithelial cell subtypes was performed using FindAllMarkers, with genes meeting the thresholds (avg_log2FC ≥ 0.5 and p_val_adj < 0.01) selected for enrichment analysis. KEGG pathway analysis demonstrated significant enrichment of cancer-related pathways in H19 + myoEPC, including the PI3K-Akt, ECM-receptor interaction, JAK-STAT, and TNF signaling pathway (Fig. 2D).

Given the predominance of malignant cells in H19 + myoEPC, NMF analysis was employed to identify the 50 most representative genes associated with this subpopulation. Furthermore, SCENIC analysis was performed to delineate the transcriptional regulatory network, with a focus on H19 + myoEpC. By intersecting the target genes of TFs with the highest RSS and the top 50 genes from the NMF-derived H19 + myoEPC program, four critical TFs were identified, including HMGA2, WT1, IRX4, and RXRA (Fig. 2E). GO and KEGG analyses of these regulators revealed significant enrichment in pathways such as JAK-STAT signaling, AGE-RAGE signaling, Rap1 signaling, and MAPK signaling. Additionally, these TFs were implicated in biological processes, including extracellular matrix organization and extracellular structure organization (Supplementary Fig. S2).

### H19 + myoEpC represent a distinctive cellular subtype in ACCB

**Fig. 3:**
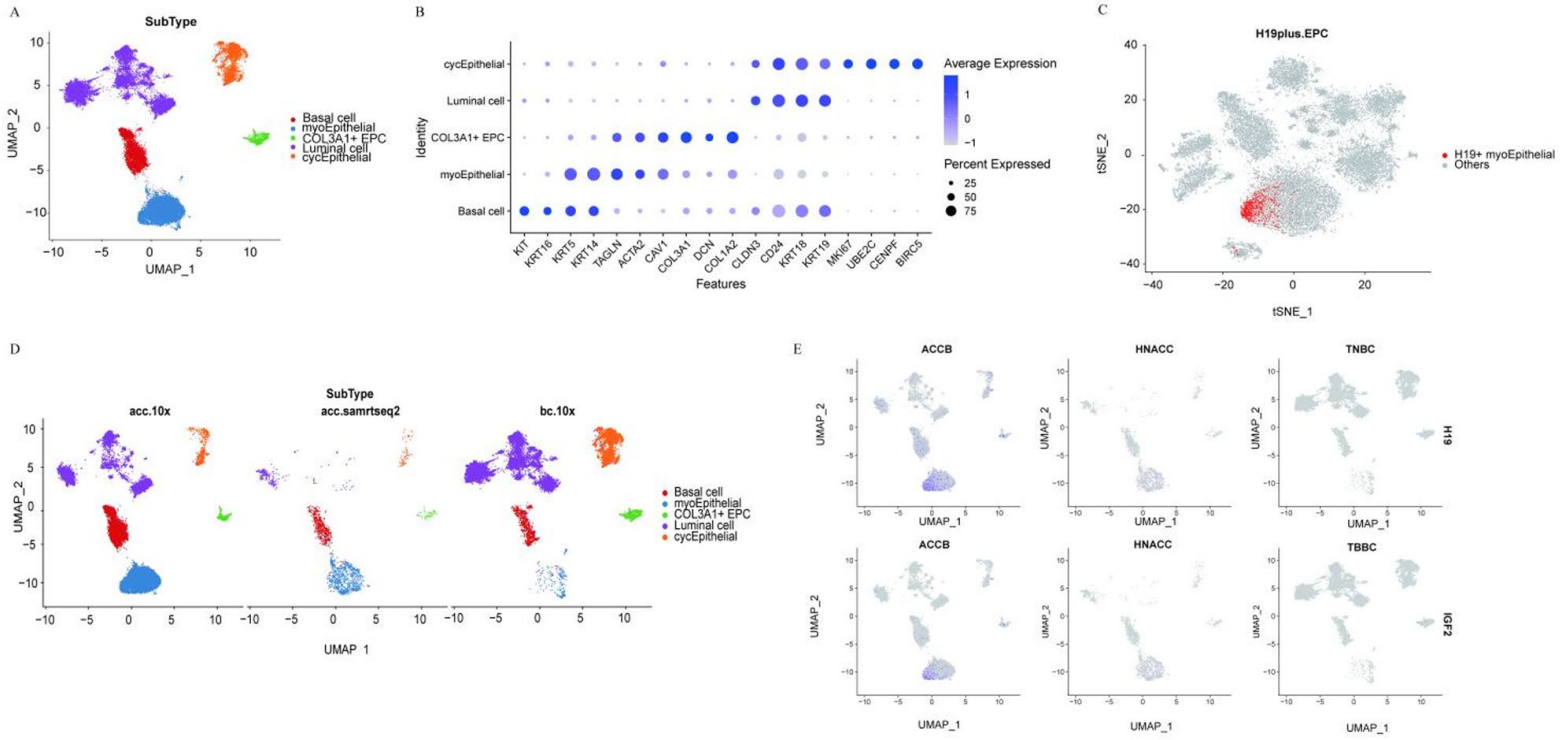
Characterization of H19 + myoEpC in ACCB. **A** Integrated UMAP projection of epithelial cells derived from ACCB, HNACC, and TNBC datasets, uniformly annotated into five shared subtypes. **B** Dot plot illustrating the expression of canonical marker genes used for the consistent annotation of epithelial subtypes across the three cancer types. **C** UMAP visualization highlighting the spatial localization of H19 + myoEpC cells (labeled in red) within the integrated epithelial landscape, showing their specific distribution within the myoepithelial compartment. **D** UMAP plots comparing the distribution of H19 + myoEpC cells across ACCB, HNACC, and TNBC samples. **E** UMAP visualization comparing the expression level of H19 + myoEpC markers (*H19* and *IGF2*) in ACCB, HNACC, and TNBC

To investigate the potential specificity of H19 + myoEpC in this ACCB sample, we performed integrated analysis combining our sequencing data with publicly available datasets of HNACC (GSE210171) and TNBC (GSE176078). Following dimensionality reduction and clustering with uniform cell type annotation, epithelial cells from all datasets were consistently classified into five major subtypes: basal cells, myoepithelial cells, COL3A1 + myoepithelial-like cells (COL3A1 + EPC), luminal cells, and cycling epithelial cells (cycEpithelial) (Fig. 3A, B). We labeled H19 + myoEpC cells using marker genes, and the results revealed that these cells were primarily localized to the left end of the myoepithelial cell population(Fig. 3C). We conducted a comparative analysis of H19 + myoEpC cell enrichment in HNACC, TNBC, and ACCB. The results demonstrated a striking predominance of H19 + myoEpC cells in ACCB, while their presence in HNACC and TNBC was minimal(Fig. 3D). To further validate the specificity of H19 + myoEpC in ACCB, we examined the expression patterns of two characteristic marker genes, *H19* and *IGF2*, in this cellular subtype. The results showed significantly elevated co-expression of *H19* and *IGF2* exclusively in H19 + myoEpC from ACCB, with H19 + IGF2+ double-positive cells uniquely restricted to this tumor type (Fig. 3E). These comprehensive findings suggest that H19 + myoEpC as a potential tumor-specific cellular signature of ACCB.

### Characterization of tumor-associated fibroblast heterogeneity in ACCB

**Fig. 4:**
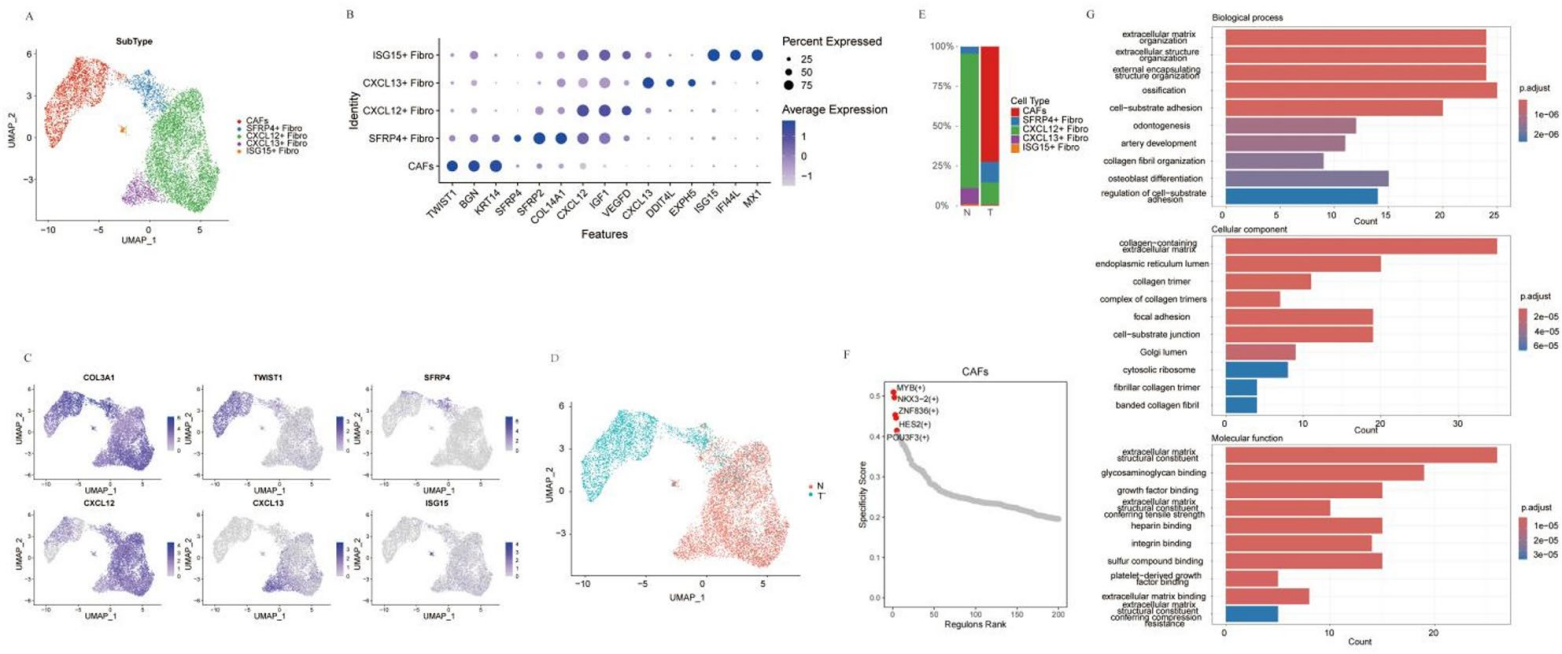
Characterization of tumor-associated fibroblast heterogeneity in ACCB. **A** UMAP plot visualizing five transcriptionally distinct fibroblast subpopulations identified from integrated tumor and adjacent normal tissues. **B** Dot plot showing the expression of marker genes for identified fibroblast subtypes. **C** UMAP plots showing the expression of specific genes in fibroblast subtypes. **D** UMAP analysis demonstrates the Fibroblast cell subtypes in tumor (T) and adjacent normal tissues (N). **E** Bar chart of fibroblast cell subtype proportions in tumor and adjacent normal tissues. **F** Plots showing the specificity scores of TFs in CAFs and a heatmap of differentially expressed genes in fibroblast subtypes. **G** GO enrichment analysis of differentially expressed genes among fibroblast subtypes. The bar chart displays selected significantly enriched terms

Comprehensive analysis of fibroblast subpopulations was performed using matched tumor and adjacent normal tissues. Unsupervised clustering identified five distinct fibroblast subtypes: CAFs, immunosuppressive CXCL12 + fibroblasts, CXCL13 + fibroblasts, and ISG15 + fibroblasts, characterized by high expression of interferon-stimulated genes (Fig. 4A, B). Notably, the CAF subpopulation exhibited marked upregulation of *KRT5*, *KRT14*, and the EMT-related gene *TWIST1*, demonstrating classical epithelial-mesenchymal transition features (Fig. 4C). We analyzed the enrichment of CAFs in adjacent normal and tumor tissues. The results showed that CAFs were exclusively derived from tumor samples, while CXCL13 + fibroblasts, which have been reported to promote tertiary lymphoid structure formation [20], predominantly originated from adjacent normal samples(Fig. 4D and E). In contrast, SFRP4 + fibroblasts and CXCL12 + fibroblasts, known to to be involved in tumor progression and metastasis [21, 22], were present in both tumor and paracancerous tissues. Transcription factor profiling identified significant enrichment of oncogenic regulators (HES2, MYB, MEF2C, and NKX3-2/BAPX1) within the CAFs (Fig. 4F).

Functional annotation of fibroblast subpopulations was performed using differential gene expression analysis (FindAllMarkers, thresholds: avg_log2FC ≥ 0.5 and p_val_adj < 0.01) followed by GO enrichment analysis (Fig. 4G). The results showed that the significantly enriched pathways included extracellular matrix organization, cell-substrate adhesion, regulation of cell-substrate adhesion, and collagen fibril organization. These molecular processes collectively contribute to EMT activation and subsequent tumor progression.

### Comprehensive profiling of immune cell subsets in the tumor microenvironment

**Fig. 5:**
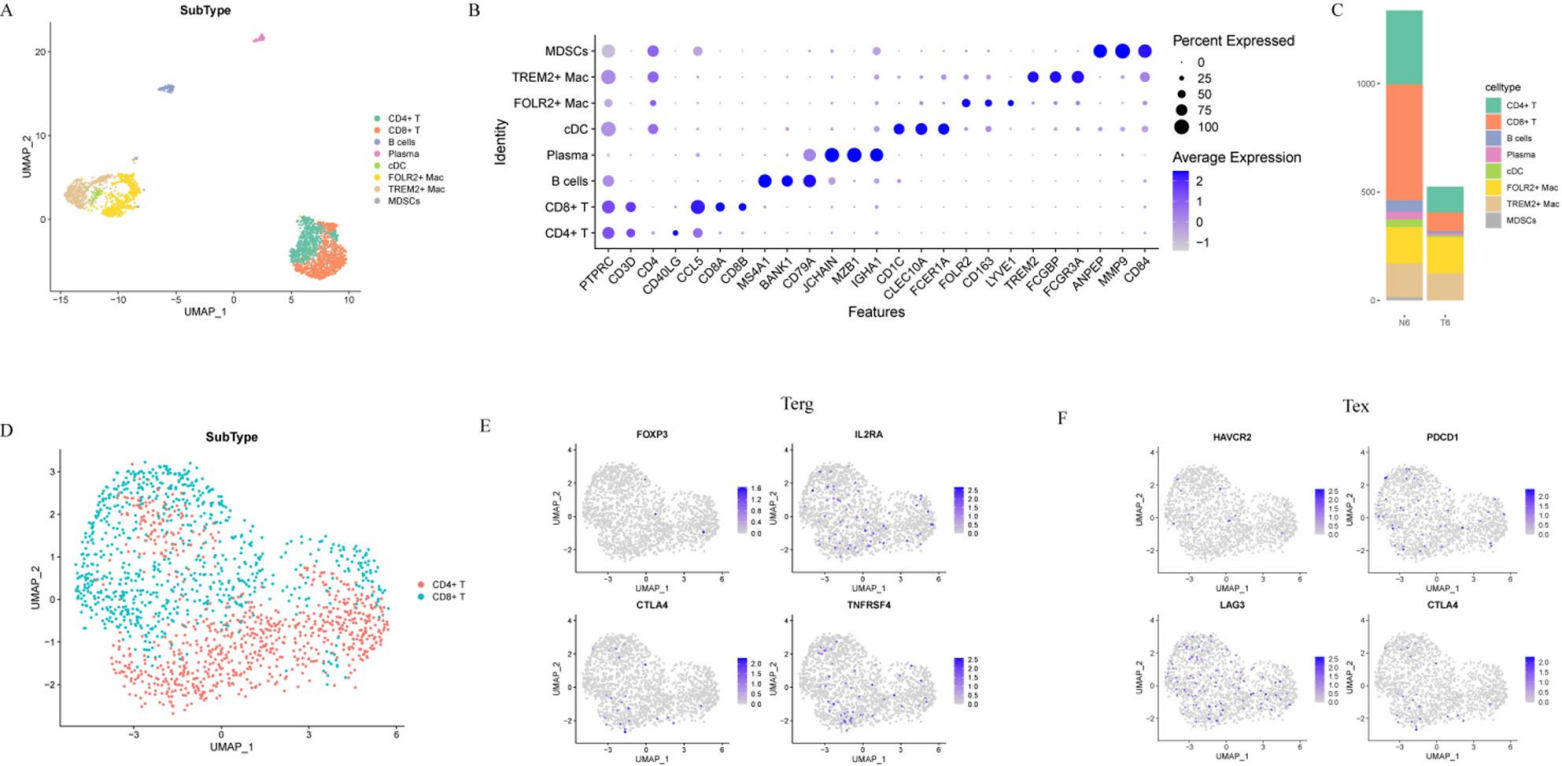
Comprehensive profiling of immune cell Subsets in the tumor microenvironment. **A** UMAP visualization of eight transcriptionally distinct immune cell subtypes identified from integrated ACCB tumor and adjacent normal tissue samples. **B** Dot plot displaying the expression levels of canonical marker genes used to annotate each immune cell subtype (dot size = percentage of cells expressing the gene; color intensity = average expression level). **C **Bar chart comparing the relative abundance of each immune cell subset between ACCB tumor and adjacent normal tissues. **D** UMAP visualization of two distinct T-cell subclusters identified through sub-clustering analysis of the merged CD4 + and CD8 + T cell populations. UMAP plots showing the expression of Treg (**E**) and Tex (**F**) marker genes across immune cell subtypes

Through systematic annotation of immune cell populations, we identified eight major subtypes: CD4 + T cells, CD8 + T cells, B cells, plasma cells, conventional dendritic cells (cDCs), FOLR2 + macrophages (FOLR2 + Mac), TREM2 + macrophages (TREM2 + Mac), and myeloid-derived suppressor cells (MDSCs) (Fig. 5A, B). To delineate immune landscape differences between tumor and adjacent normal tissues microenvironments, we normalized cell counts per sample (10,000 cells/sample) and compared immune subset abundances. The results revealed significantly reduced infiltration of CD4 + T cells, CD8 + T cells, and B cells in tumor tissues compared to adjacent normal tissues, indicating an immunosuppressive tumor microenvironment (Fig. 5C). To explore the potential mechanisms driving immunosuppression, we performed a combined extraction of the CD4 + T cell and CD8 + T cell populations identified above, conducted sub-clustering analysis on the merged T cells, and UMAP visualization revealed distinct T cell subpopulations (Fig. 5D). Notably, the expression levels of marker genes for regulatory T cells (Tregs) and exhausted T cells (Tex) were minimal across all immune subsets (Fig. 5E, F), indicating the absence of classical Treg and Tex populations. These results suggest that the observed immunosuppressive state may be predominantly driven by non-cell-autonomous mechanisms rather than the conventional paradigms of T cell dysfunction.

### Intercellular communication networks of H19 + myoEpC drive tumor progression and immunosuppressive

**Fig. 6:**
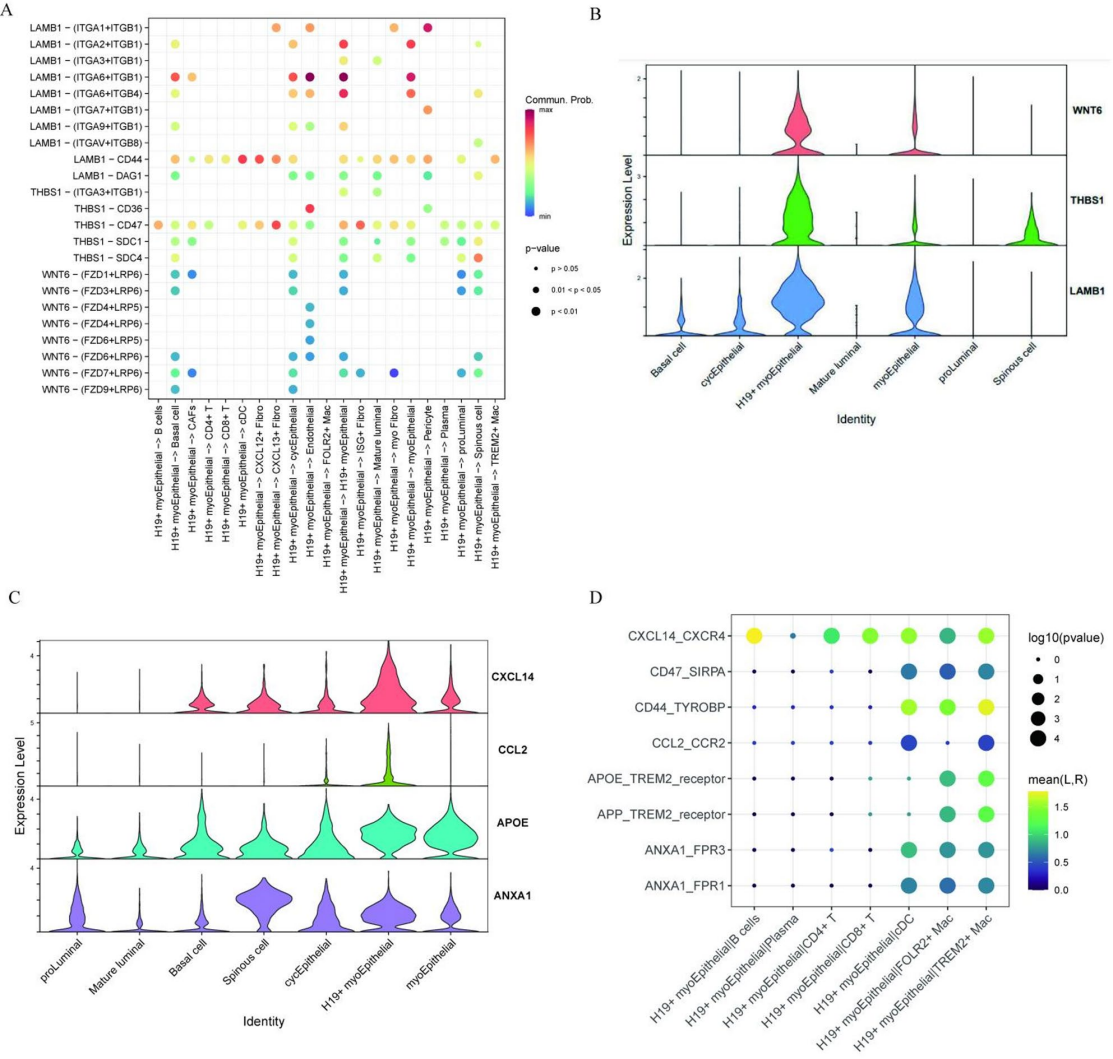
The interaction networks of H19 + myoEpC with tumor microenvironment Cells. **A** Network diagram illustrating the dominant ligand-receptor interactions initiated by H19 + myoEpC with major cellular compartments (fibroblasts, epithelial cells, and immune cells) in the ACCB microenvironment. **B** Violin plots comparing the expression levels of key signaling ligands in H19 + myoEpC versus other epithelial cell subclusters, demonstrating their specific overexpression in this malignant subpopulation. **C** Violin plots comparing the expression levels of immunomodulatory ligands (CXCL14, CCL2, APOE, ANXA1) between H19⁺ myoepithelial cells and other epithelial subtypes. **D** Ligand-receptor interaction heatmap, depicting the predicted interaction strength and statistical significance between immunomodulatory ligands expressed by H19⁺ myoepithelial cells and receptors expressed by myeloid cells (particularly macrophages) within the tumor microenvironment

To elucidate the mechanisms underlying both immunosuppression and tumor progression in ACCB, we performed comprehensive cell-cell interaction analysis focusing on the tumor-specific H19 + myoEpC subpopulation. Our results demonstrate that H19 + myoEpC engages in extensive crosstalk with CAFs, conventional myoepithelial cells, and endothelial cells primarily through LAMB-(ITGA6 + ITGB1) ligand-receptor pairs (Fig. 6A). Notably, H19 + myoEpC interacts with T cells via the LAMB1-CD44 axis, while exhibiting prominent LAMB1-CD47 signaling with fibroblastic populations. Furthermore, WNT6-(FZD9 + LRP6)-mediated interactions dominate the communication between H19 + myoEpC and both epithelial cells and CAFs. Expression profiling revealed aberrant overexpression of key ligands_including WNT, THBS1, and LAMB1 - specifically in the H19 + myoEpC subpopulation compared to other epithelial subtypes (Fig. 6B). These findings establish that H19 + myoEpC orchestrates a multifaceted signaling network through paracrine interactions with CAFs, immune cells, and epithelial compartments. To further elucidate the active regulatory role of H19⁺ myoepithelial cells in shaping the immunosuppressive microenvironment, we conducted an in-depth analysis of their specifically expressed immunomodulatory ligand profile and their potential interactions. The results demonstrated that, compared to all other epithelial subtypes, H19⁺ myoepithelial cells significantly overexpress a panel of key ligands targeting myeloid immune cells, including the chemokines CXCL14 and CCL2, apolipoprotein APOE, and annexin ANXA1 (Fig. 6C). Ligand-receptor interaction-based cell communication analysis further confirmed that these ligands exhibit a series of significantly enriched interactions with corresponding receptors highly expressed on myeloid cells—particularly macrophages—within the tumor microenvironment (Fig. 6D). Among these, CXCL14-CXCR4, CCL2-CCR2, and CD47-SIRPA showed high statistical significance. Particularly noteworthy is that H19⁺ myoepithelial cells express the immune checkpoint molecule CD47, and its predicted binding to the macrophage receptor SIRPA strongly suggests that this cell subpopulation may directly inhibit the phagocytic function of macrophages, thereby promoting immune evasion. Moreover, we found that H19 + myoEpCs also exhibited the highest expression level of interleukin-20 (IL-20) among all cell types in the ACCB microenvironment (Supplementary Fig. S3).

## Discussion

Based on scRNA-seq analysis of a single case, this study provides an initial dissection of the cellular heterogeneity, malignant cell evolution characteristics, and dynamic interaction network of the tumor microenvironment in ACCB. It revealed the core role and regulatory mechanisms of H19 + myoEpC as a specific malignant subpopulation in ACCB. Compared with HNACC and TNBC, H19 + myoEpC are significantly enriched in this case of ACCB and specifically express the H19/IGF2 double-positive marker, suggesting that they may serve as a cellular identity marker for ACCB. Further analysis revealed that H19 + myoEpC activate canonical oncogenic pathways such as PI3K-Akt and JAK-STAT, and abnormally overexpress ligand molecules, including LAMB1 and WNT6, forming multidimensional interaction networks with CAFs, endothelial cells, and immune cells. This finding not only provides new evidence for the molecular classification of ACCB but also reveals its unique malignant drive mode.

In this study, we employed scRNA-seq to comprehensively analyze the cellular landscape of a single case of ACCB and its adjacent normal tissues, revealing the cellular composition and heterogeneity of the ACCB microenvironment. The investigation of cellular heterogeneity and tissue-specific distribution patterns indicates that significant cellular reorganization occurs in ACCB. The enrichment of specific cell types or phenotypes in tumor tissues compared to adjacent normal tissues may reflect adaptive changes that support tumor progression and immune evasion. Furthermore, the presence of fibroblasts and myeloid cells (such as TREM2 + macrophages) in the tumor microenvironment suggests their potential role in modulating the immune landscape. Our findings demonstrate that CAFs secrete CXCL12, which contributes to T cell exclusion and immune evasion [23]. Additionally, the interaction between myoEpC and TREM2 + macrophages via the THBS1-CD47 axis may further impair immune cell function.

This study identified myoEpC as a unique cellular subtype in this case of ACCB, highlighting its potential as a tumor-specific marker. The selective enrichment of H19 + myoEpC in ACCB, and their minimal presence in other cancer types such as HNACC and TNBC, underscores the distinct cellular landscape of ACCB. In addition, there was significant functional heterogeneity of fibroblasts in ACCB. The CAF subpopulation specifically overexpressed EMT-related genes (such as TWIST1 and KRT5/KRT14) and formed tight interactions with H19 + myoEpC via signaling axes, such as LAMB1-CD47. Notably, CXCL12 + fibroblasts with immune-suppressive features are widely present in both tumor and adjacent normal tissues, while CXCL13 + fibroblasts that promote the formation of tertiary lymphoid structures are mainly enriched in the adjacent normal tissues. This distribution pattern may explain the persistence of the immunosuppressive state in the ACCB microenvironment [24, 25]. In addition, the abnormal activation of TFs HES2 and MYB in CAFs suggests the potential regulatory roles of Notch and MYB signaling pathways in fibroblast functional polarization [26]. The above findings indicate the significant role of the MYB signaling network in the occurrence and development of ACCB.

In the immune microenvironment of this ACCB case, ACCB exhibited a significant reduction in the infiltration of CD4+/CD8 + T cells and B cells. However, unlike traditional immune-suppressive tumors, the expression of Treg and exhausted T cell (Tex) signature genes was absent. This suggests that the immune evasion of ACCB may rely on non-canonical mechanisms. Combined with cell interaction analysis, the signaling pathways mediated by H19 + myoEpC and myeloid cells (such as TREM2 + macrophages) through THBS1-CD47, as well as the T cell exclusion effect mediated by CXCL12 secreted by CAFs, may be the key factors leading to immune cell dysfunction [27]. This finding suggests new ideas for the immunotherapy of ACCB, intervention strategies targeting myeloid or stromal cells rather than T cells themselves may hold greater potential.

Comprehensive analysis at the single-cell level revealed ACCB, HNACC, and TNBC to share four main features, suggesting their different immunogenicity and therapeutic response profiles promoted by their intrinsic cellular cultural milieu and transcriptomic signature. The H19 + myoEpC, which belong to the ACCB-specific malignant subpopulation, significantly upregulated ligands related to myeloid chemoattraction and polarisation. Notable among these ligands were CD47, CXCL14, CCL2, APOE, and ANXA1. The CD47-SIRPA axis is a thoroughly studied immune checkpoint that inhibits macrophage phagocytosis [28]. Moreover, CXCL14, CCL2, APOE and ANXA1 have been shown to promote monocyte recruitment and M2-like polarisation [29–31]. The findings indicate that ACCB malignant cells have the intrinsic capability to sculpt a myeloid-based immunosuppressive microenvironment. At the same time, ACCB CAFs exhibited a selective increase in CXCL12 and TWIST1, which are crucial factors involved in T cell exclusion and epithelial-mesenchymal transition, respectively [32, 33]. CXCL12 overexpression has been associated with the immune desert phenotype in a variety of cancers, which could explain low levels of Treg and Tex infiltration in ACCB. The ACCB microenvironment also had TREM2 + TAMs, which engaged in a THBS1-CD47 interaction axis with H19 + myoEpC. TREM2 + TAMs are known to be mediators of immunosuppression and anti-PD-1 therapy resistance [34]. This myeloid-centric immunosuppressive architecture is in stark contrast to the conventional T cell exhaustion-dominated environment typical for TNBC. Importantly, T cells in ACCB express little to no FOXP3, PDCD1, or TOX. Thus, exclusion of immune cells, rather than induction of T cell exhaustion or recruitment of regulatory T cells, is how this tumor evades the immune response. These findings hold important implications for rational therapeutics in ACCB. Next, while PD-1/PD-L1 targeted immune checkpoint inhibitors may offer poor efficacy, therapeutic strategies targeting the CXCL12-CXCR4, CD47-SIRPA, and macrophage polarization are more promising strategies.

This study reveals that H19+ myoepithelial cells, specifically enriched in this case of ACCB, highly express a panel of key ligands known to drive macrophage polarization toward the M2 phenotype, including CXCL14, CCL2, APOE, and ANXA1. This finding provides mechanistic insight into the unique “myeloid-centric” immunosuppressive landscape characteristic of ACCB. The existing literature indicates that the CXCL14-CXCR4 signaling axis promotes M2 macrophage migration and polarization and participates in immune regulation by activating the NF-κB pathway [35, 36]. CCL2 is a key chemokine mediating monocyte/macrophage recruitment, while both APOE and ANXA1 have been demonstrated to directly induce macrophage differentiation into an anti-inflammatory, protumorigenic M2 phenotype [37–40]. Based on this, we propose that H19⁺ myoepithelial cells may function as a “signaling hub,” synchronously activating multiple parallel pathways to cooperatively establish and maintain a tumor microenvironment enriched with M2-type tumor-associated macrophages (TAMs). This role positions them as a critical regulatory node linking tumor epithelium to the immunosuppressive microenvironment. In recent years, targeting M2-type macrophages has emerged as a key strategy to enhance the efficacy of tumor immunotherapy [41]. These findings suggest that intervening with H19⁺ myoepithelial cells themselves or their secreted key ligands may represent a highly selective therapeutic approach. By targeting this upstream regulatory nexus, it may be possible to simultaneously reverse immunosuppression and suppress tumor progression, thereby offering novel combinatorial treatment avenues for ACCB.

This study has several limitations. First, the findings are derived from a single patient sample, which inherently restricts the generalizability of the results and necessitates future validation in larger cohorts. Second, the study lacks independent experimental validation of key observations, such as the elevated expression of H19 and IGF2, and has not yet established their correlation with clinical outcomes or prognostic significance. Furthermore, the functional roles of the identified H19+ myoepithelial cells and key signaling pathways (e.g., LAMB1-CD47, WNT6-FZD9) remain to be confirmed through in vitro and in vivo experimental models. Future research should focus on validating these findings in larger, independent clinical cohorts. Despite these limitations, this study provides an important theoretical basis for the molecular mechanism dissection and targeted therapy of ACCB, a rare malignant tumor, through single-cell analysis.

## Supplementary Information

Supplementary Material 1: Violin plot of CNV scores among different subgroups.

Supplementary Material 2: Expression of interleukin-20 (IL-20) across cell types in the ACCB microenvironment.

Supplementary Material 3: GO and KEGG pathway enrichment analysis was performed on *HMGA2, WT1, IRX4*, and *RXRA*.

## Data Availability

The raw data supporting the conclusions of this article is available in the CNCB database under the accession number PRJCA053564.
