# Supplementary figures and images for: Single-cell RNA sequencing of adenoid cystic carcinoma of the breast reveals cellular heterogeneity and tumor microenvironment features
Source: BMC Med Genomics. 2026 Feb 27;19:55. doi: 10.1186/s12920-026-02329-2 (PMC13041279; doi:10.1186/s12920-026-02329-2)

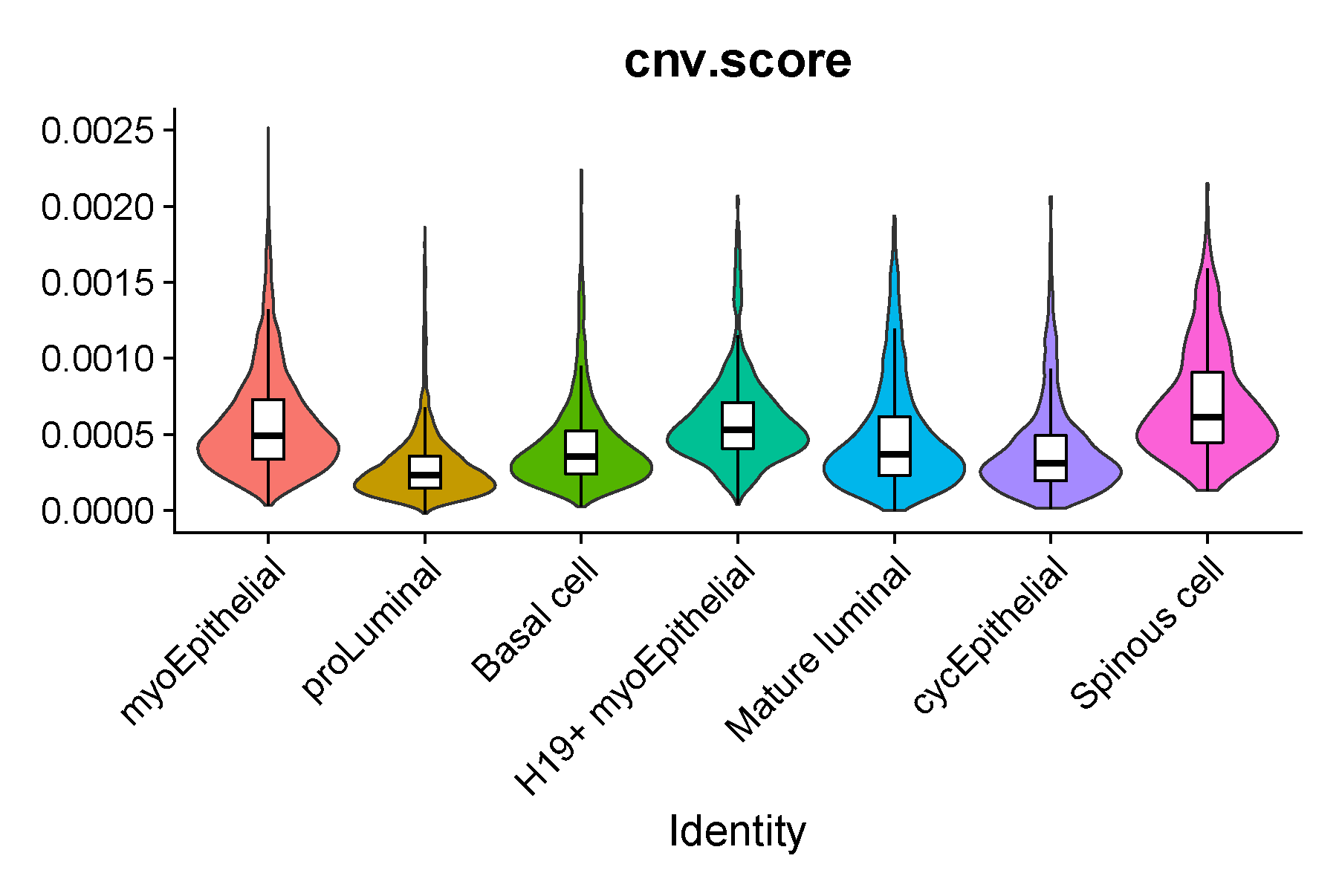

Supplement: Supplementary file 1 — Supplementary Material 1: Violin plot of CNV scores among different subgroups. [file 12920_2026_2329_MOESM1_ESM.tif]

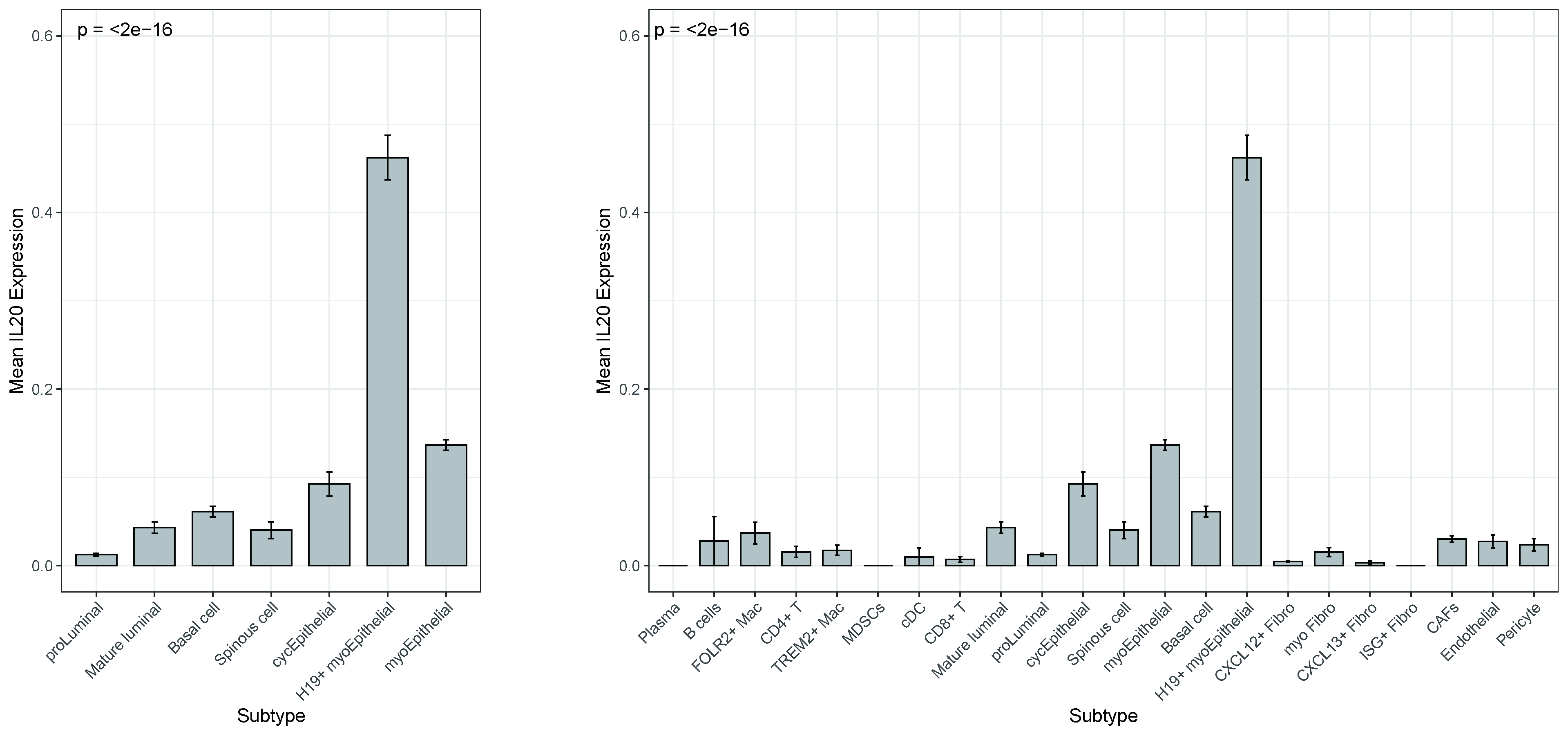

Supplement: Supplementary file 2 — Supplementary Material 2: Expression of interleukin-20 (IL-20) across cell types in the ACCB microenvironment. [file 12920_2026_2329_MOESM2_ESM.tif]
